# Supplementary figures and images for: Recovery of the maternal skeleton after lactation is impaired by advanced maternal age but not by reduced IGF availability in the mouse
Source: PLoS One. 2021 Sep 1;16(9):e0256906. doi: 10.1371/journal.pone.0256906 (PMC8409645; doi:10.1371/journal.pone.0256906)

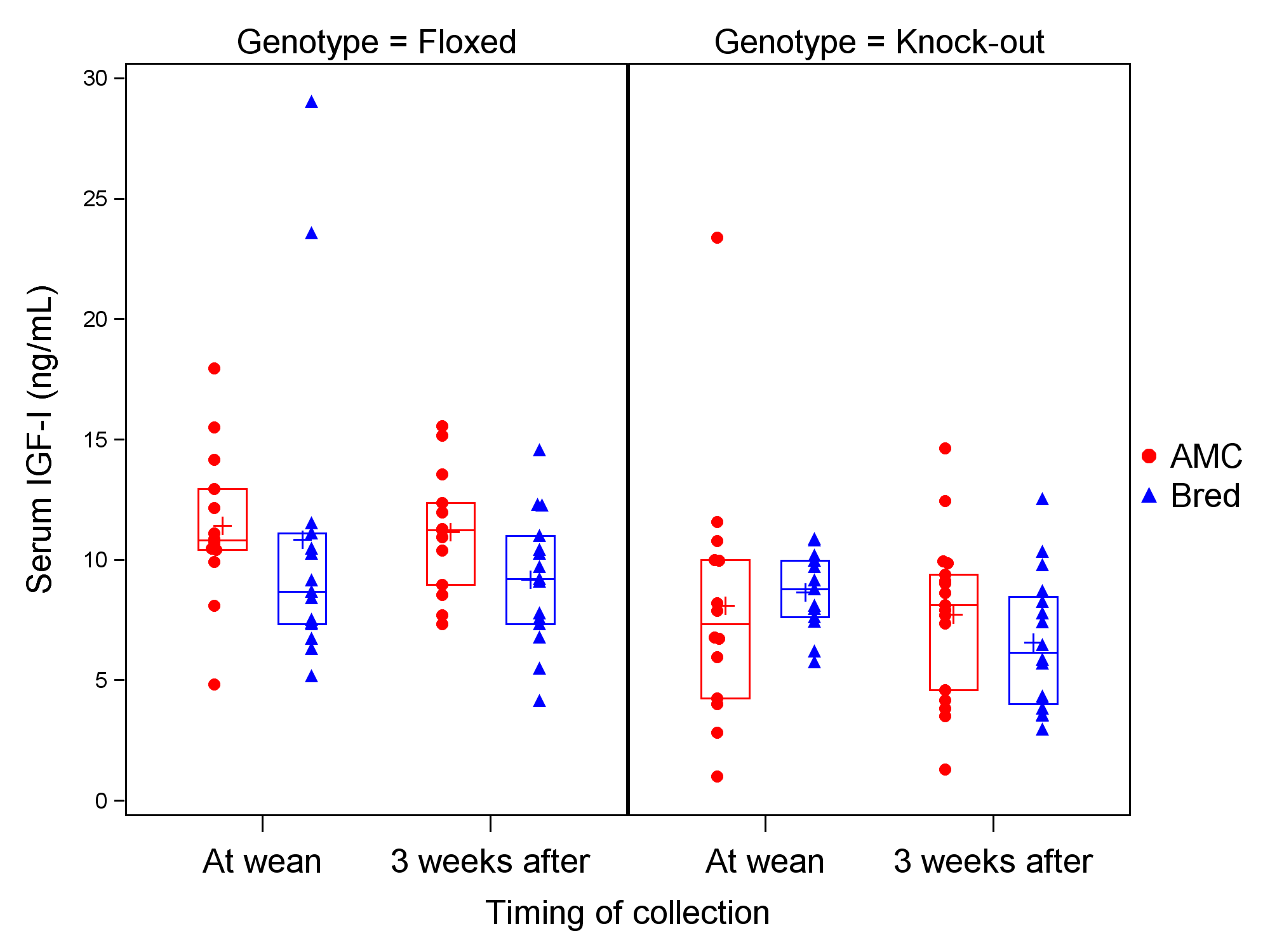

Supplement: S1 Fig — Blue triangles denote bred mice, and red circles denote age-matched controls (AMC). Crosses denote means, while horizontal lines denote the 25th, 50th and 75th percentiles. (TIF) [file pone.0256906.s001.tif]

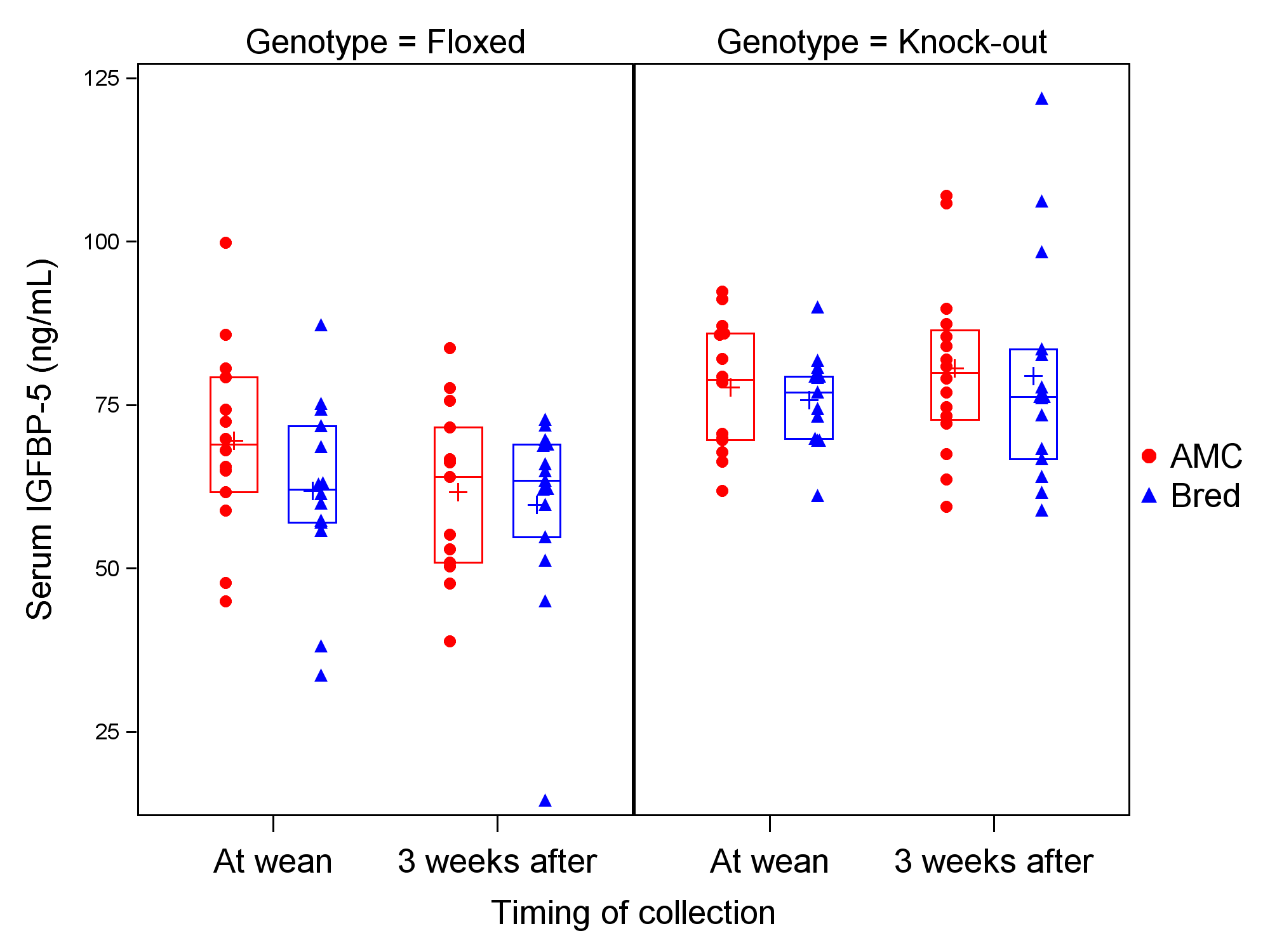

Supplement: S2 Fig — Blue triangles denote bred mice, and red circles denote age-matched controls (AMC). Crosses denote means, while horizontal lines denote the 25th, 50th and 75th percentiles. (TIF) [file pone.0256906.s002.tif]

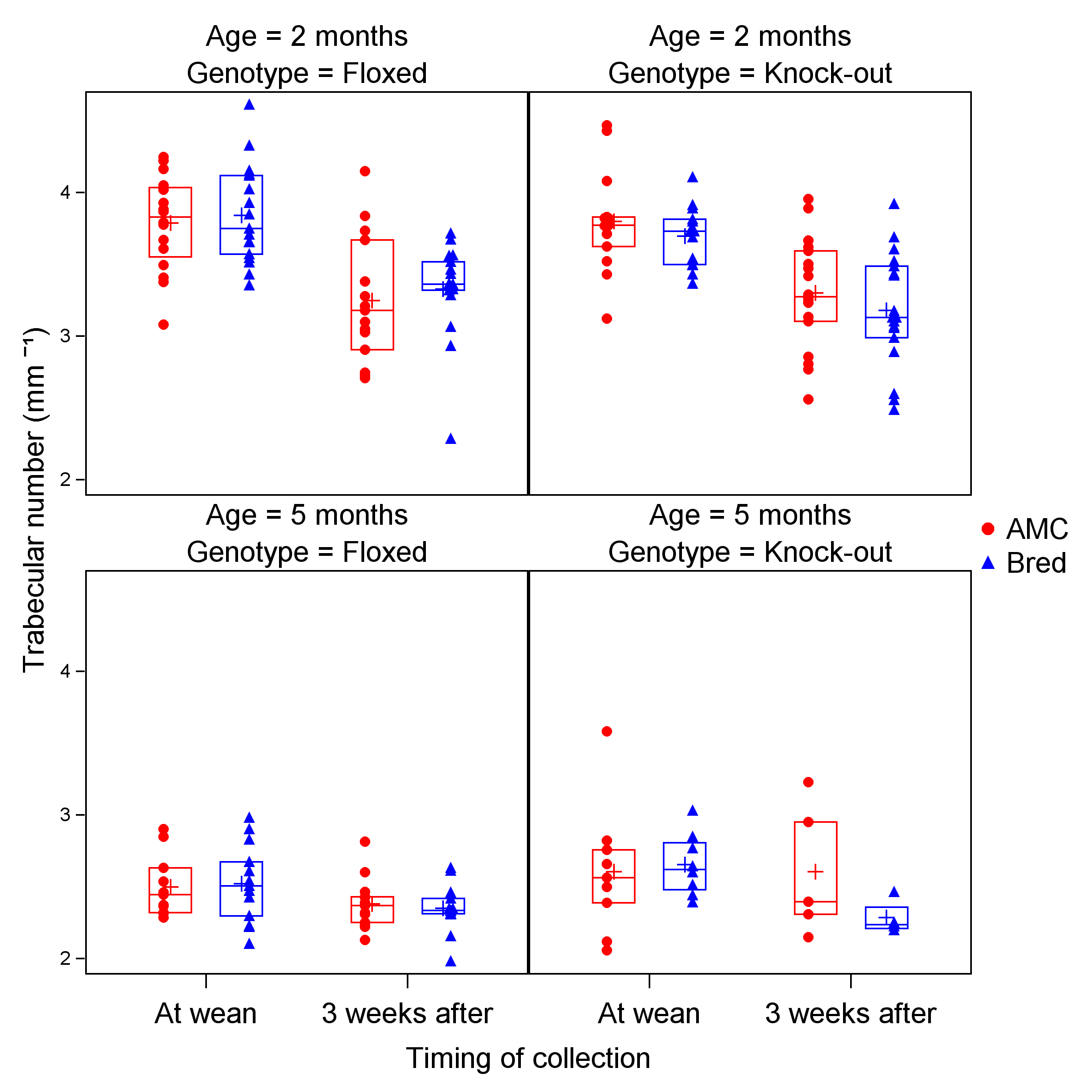

Supplement: S3 Fig — Blue triangles denote bred mice, and red circles denote age-matched controls (AMC). Crosses denote means, while horizontal lines denote the 25th, 50th and 75th percentiles. (TIF) [file pone.0256906.s003.tif]

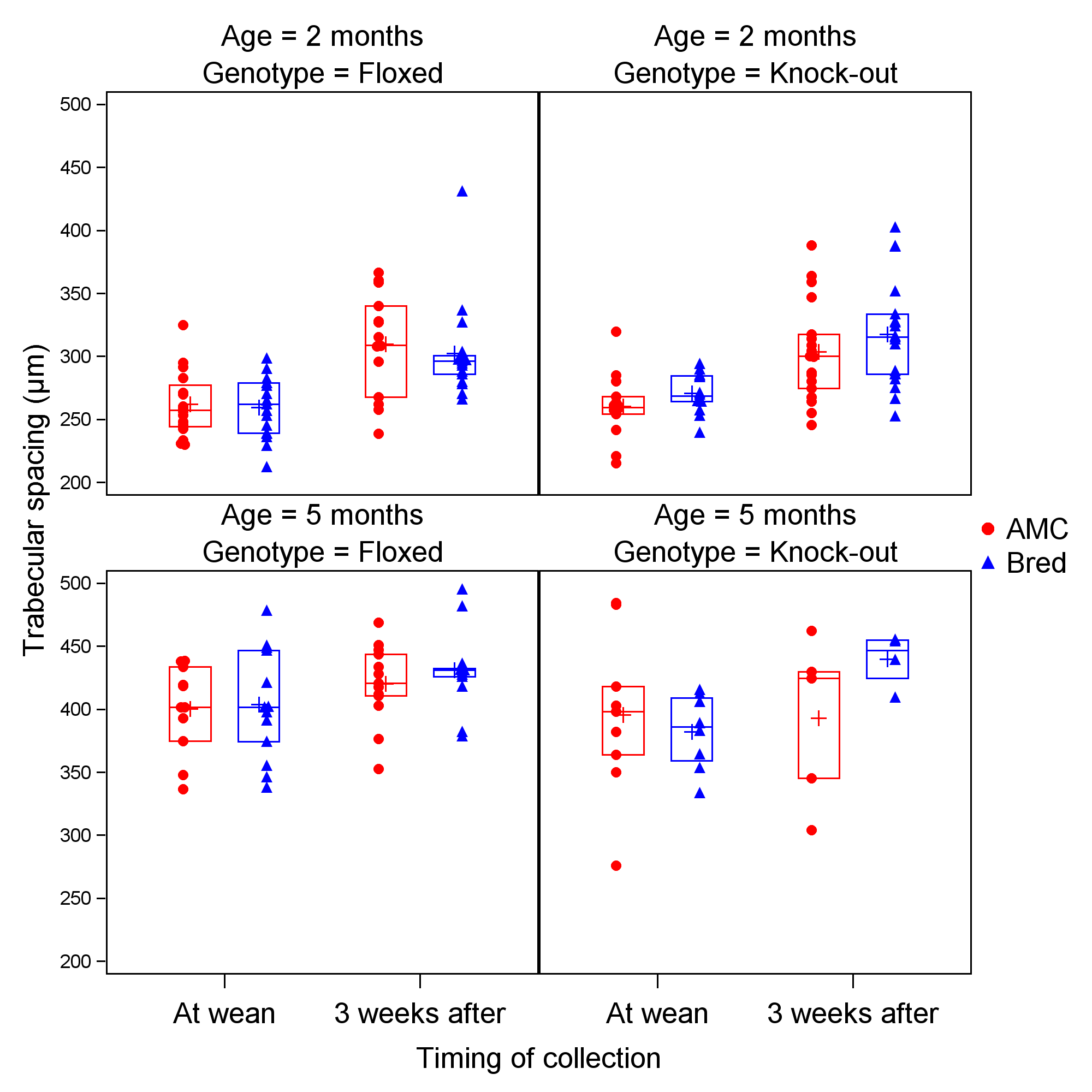

Supplement: S4 Fig — Blue triangles denote bred mice, and red circles denote age-matched controls (AMC). Crosses denote means, while horizontal lines denote the 25th, 50th and 75th percentiles. (TIF) [file pone.0256906.s004.tif]

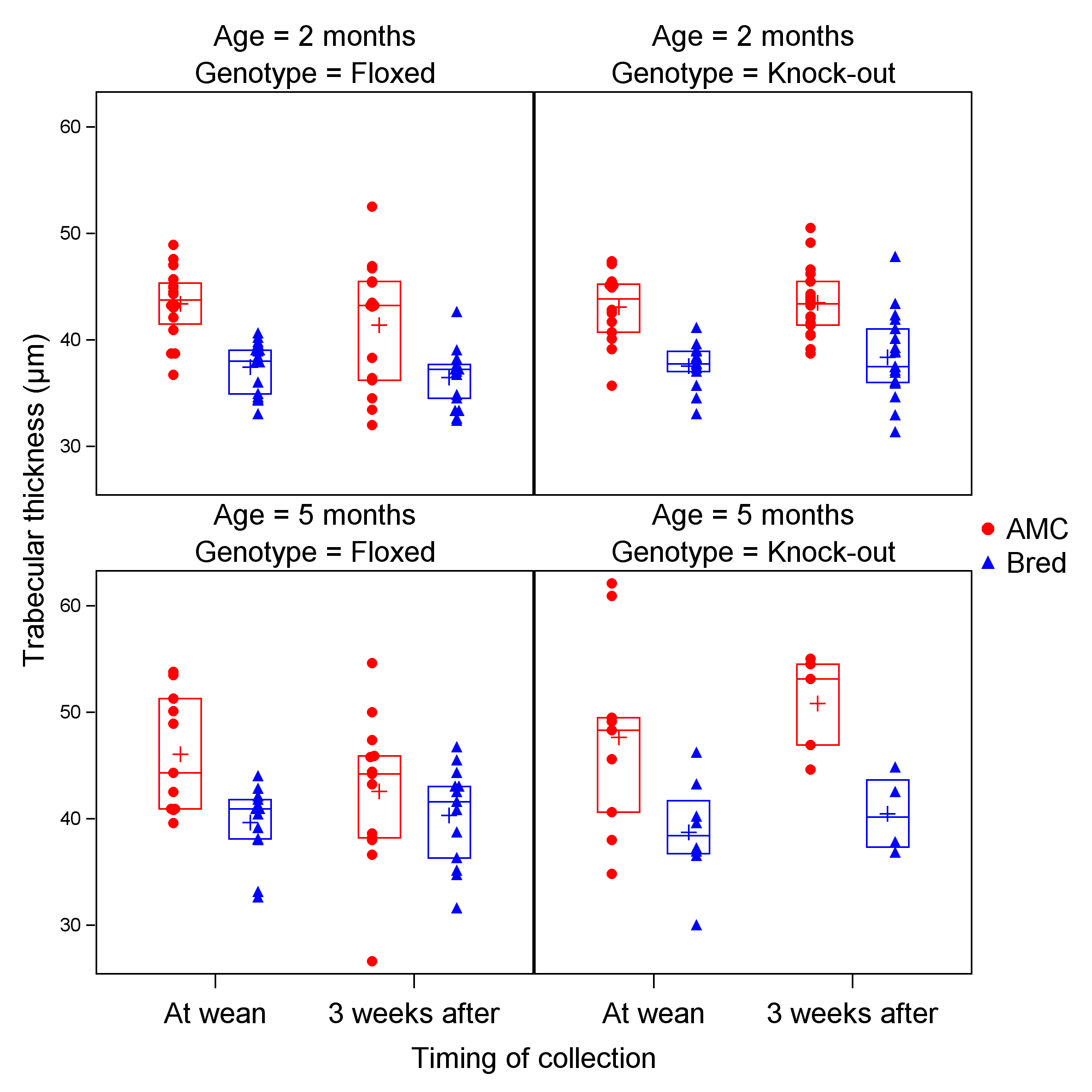

Supplement: S5 Fig — Blue triangles denote bred mice, and red circles denote age-matched controls (AMC). Crosses denote means, while horizontal lines denote the 25th, 50th and 75th percentiles. (TIF) [file pone.0256906.s005.tif]

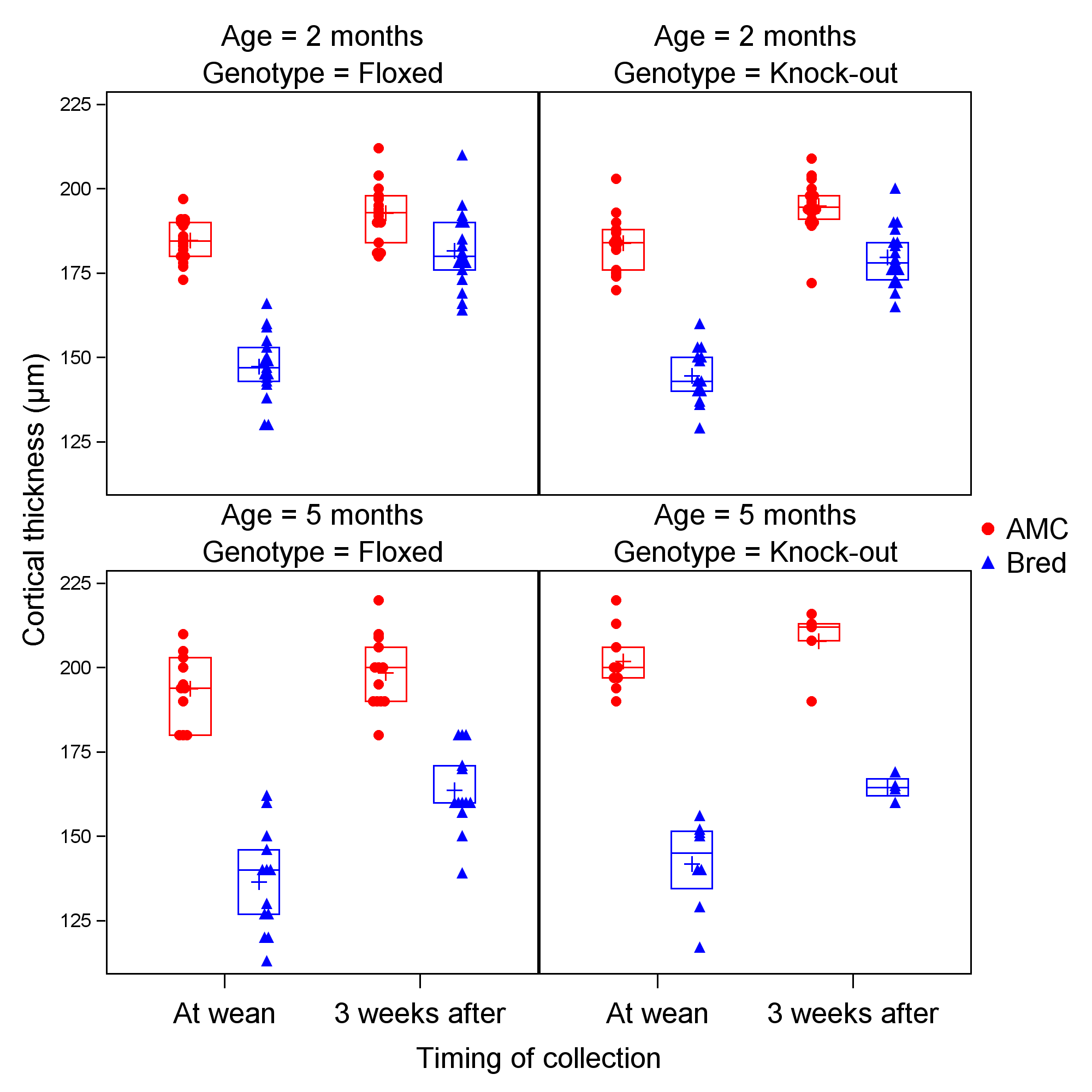

Supplement: S6 Fig — Blue triangles denote bred mice, and red circles denote age-matched controls (AMC). Crosses denote means, while horizontal lines denote the 25th, 50th and 75th percentiles. (TIF) [file pone.0256906.s006.tif]

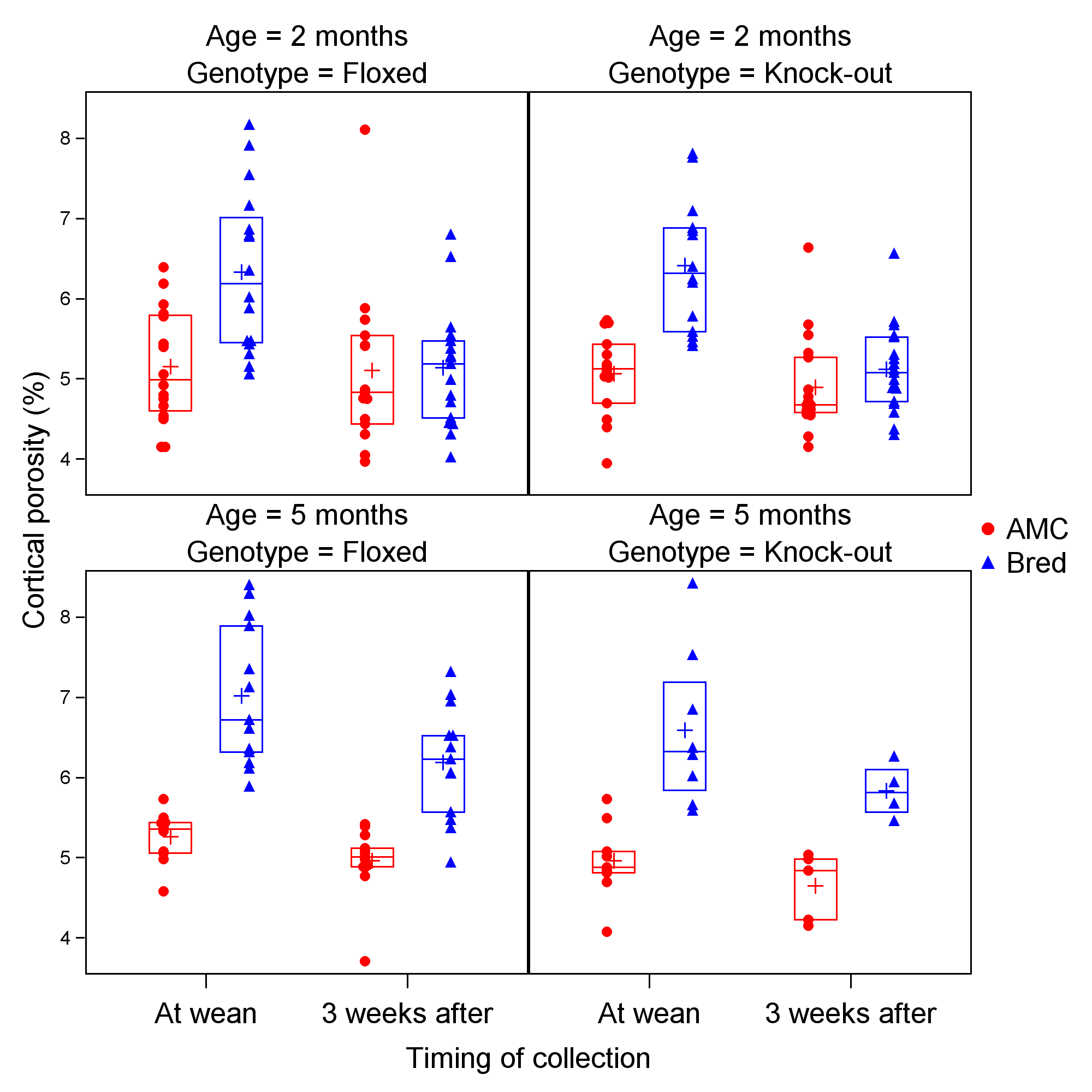

Supplement: S7 Fig — Blue triangles denote bred mice, and red circles denote age-matched controls (AMC). Crosses denote means, while horizontal lines denote the 25th, 50th and 75th percentiles. (TIF) [file pone.0256906.s007.tif]

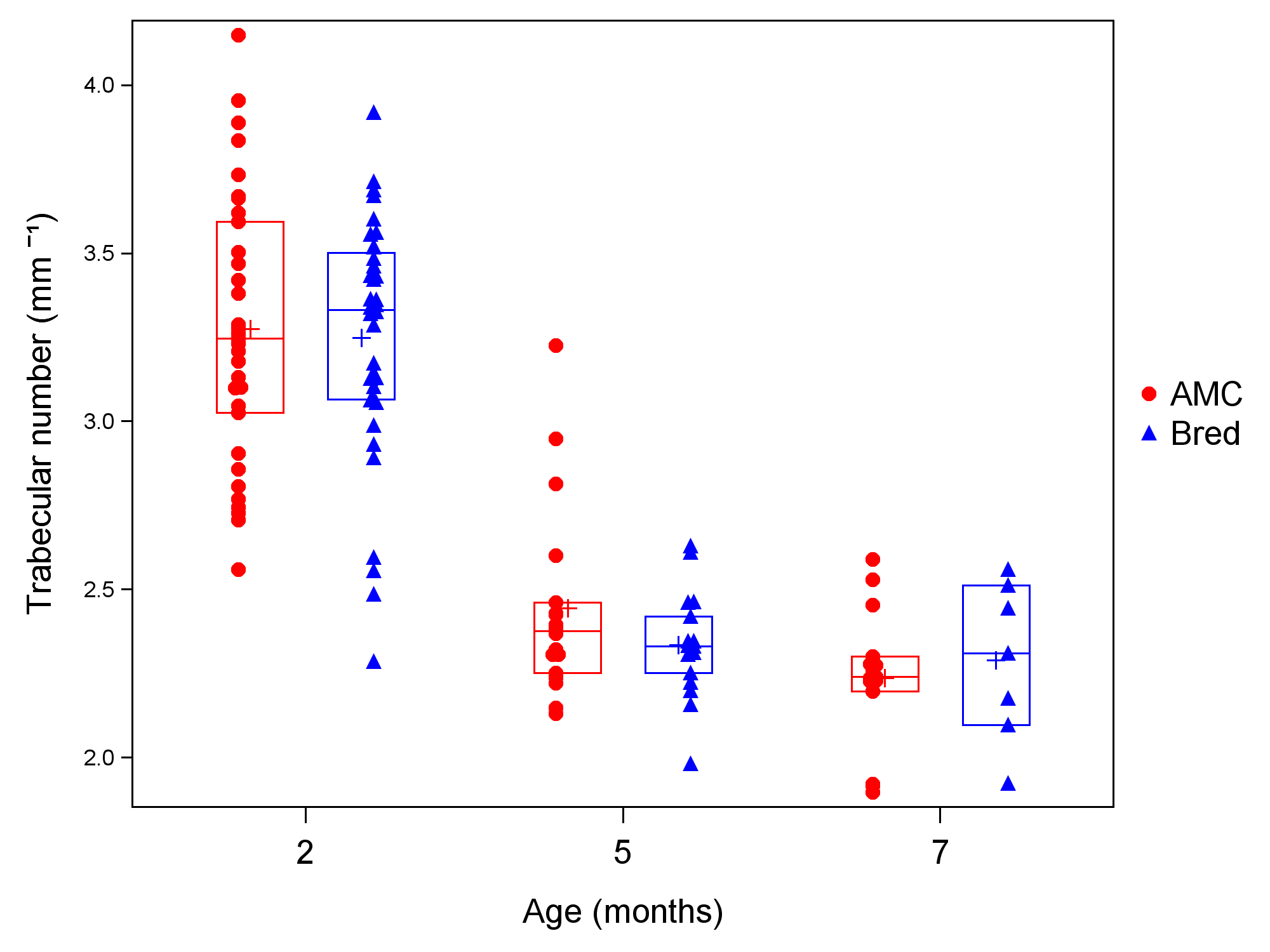

Supplement: S8 Fig — Blue triangles denote bred mice, and red circles denote age-matched controls (AMC). Crosses denote means, while horizontal lines denote the 25th, 50th and 75th percentiles. (TIF) [file pone.0256906.s008.tif]

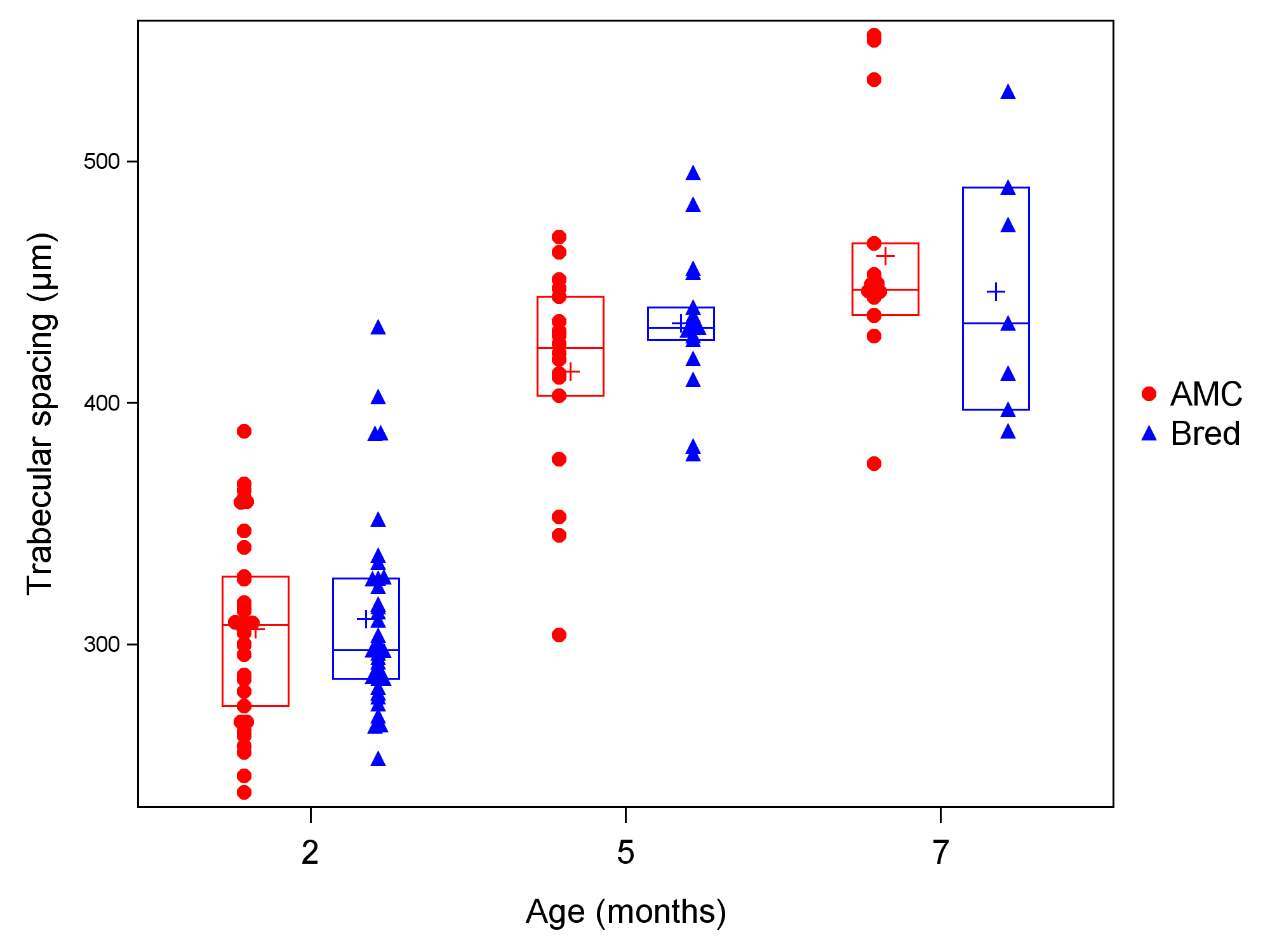

Supplement: S9 Fig — Blue triangles denote bred mice, and red circles denote age-matched controls (AMC). Crosses denote means, while horizontal lines denote the 25th, 50th and 75th percentiles. (TIF) [file pone.0256906.s009.tif]

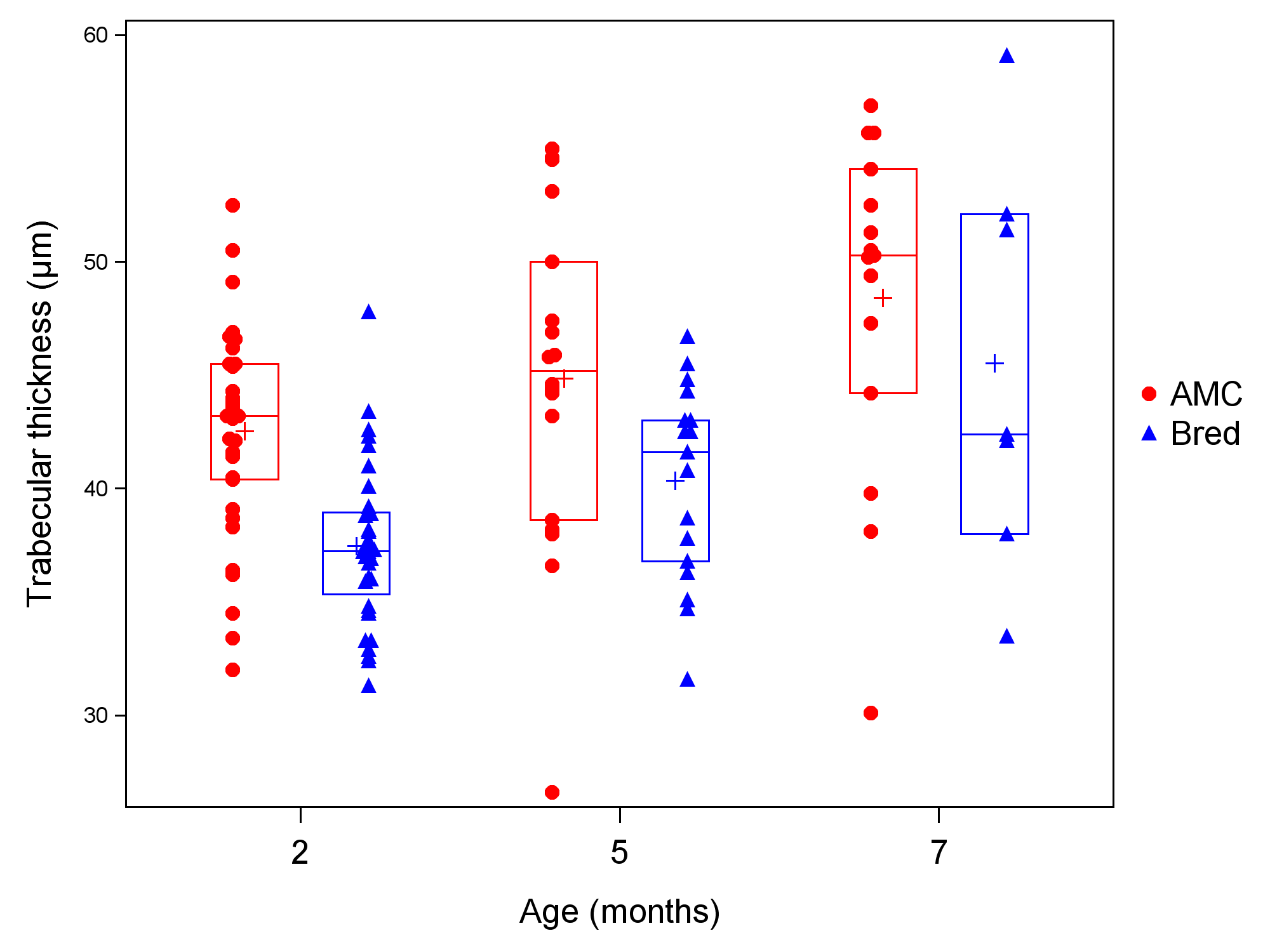

Supplement: S10 Fig — Blue triangles denote bred mice, and red circles denote age-matched controls (AMC). Crosses denote means, while horizontal lines denote the 25th, 50th and 75th percentiles. (TIF) [file pone.0256906.s010.tif]

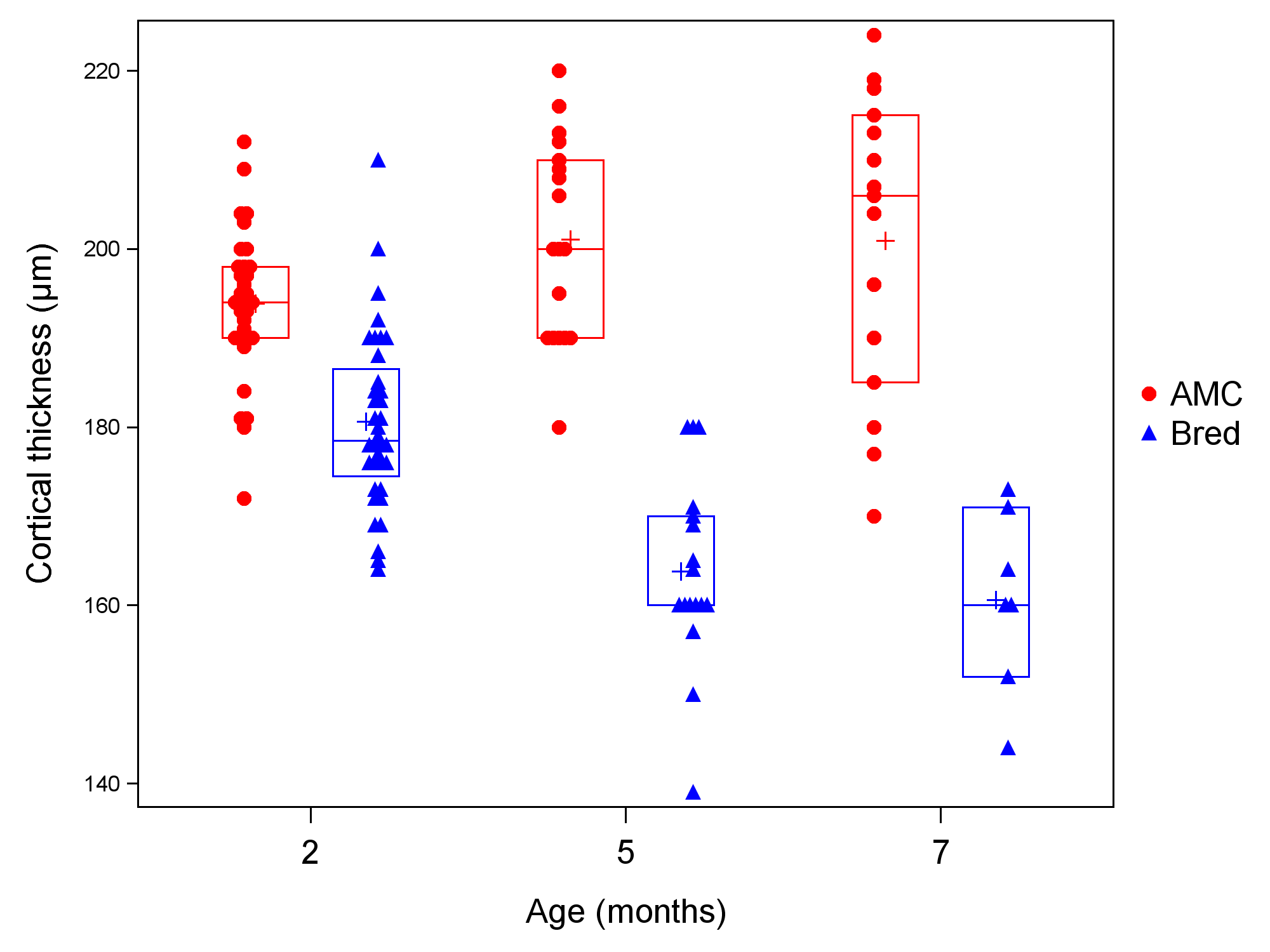

Supplement: S11 Fig — Blue triangles denote bred mice, and red circles denote age-matched controls (AMC). Crosses denote means, while horizontal lines denote the 25th, 50th and 75th percentiles. (TIF) [file pone.0256906.s011.tif]

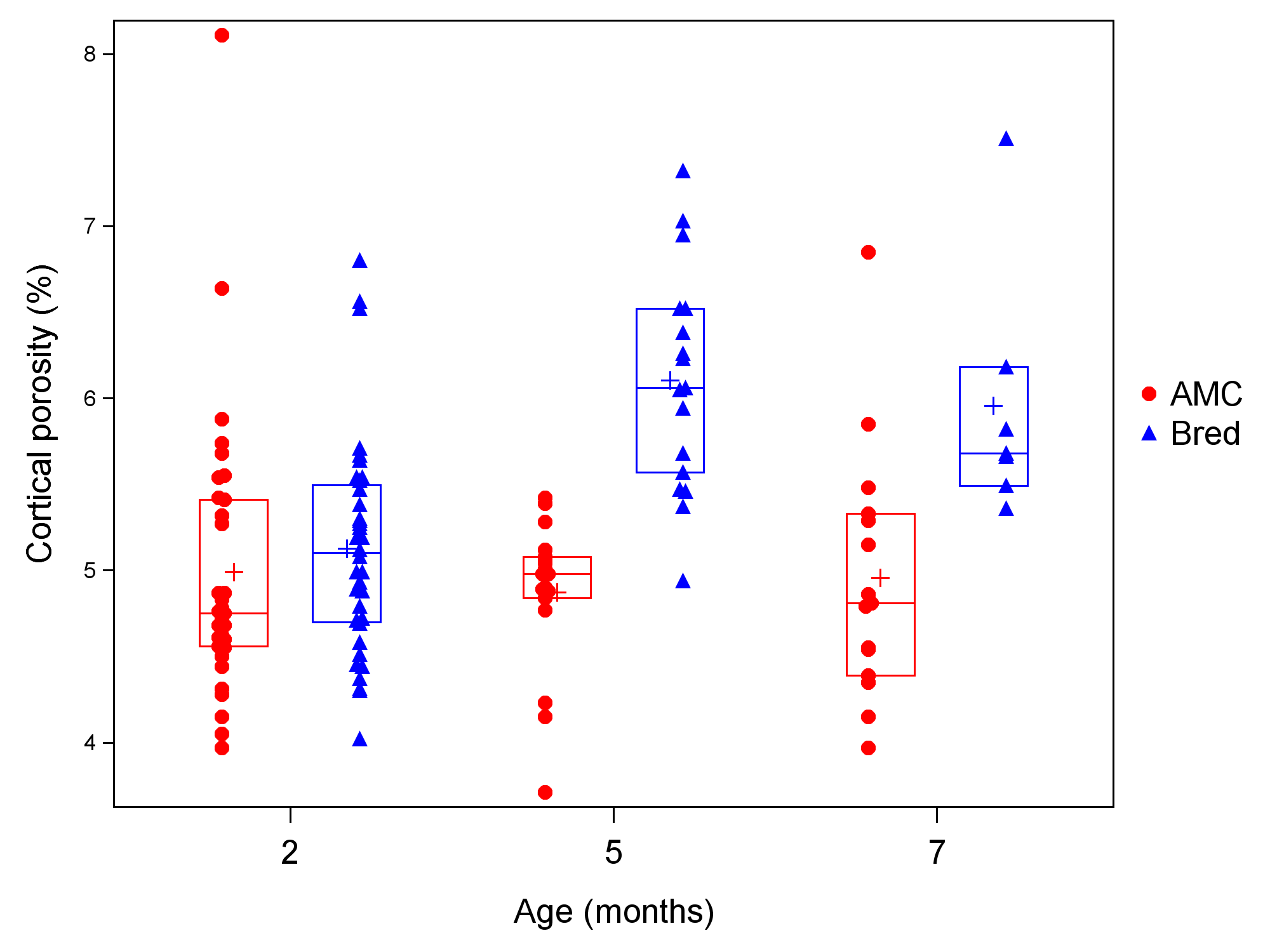

Supplement: S12 Fig — Blue triangles denote bred mice, and red circles denote age-matched controls (AMC). Crosses denote means, while horizontal lines denote the 25th, 50th and 75th percentiles. (TIF) [file pone.0256906.s012.tif]

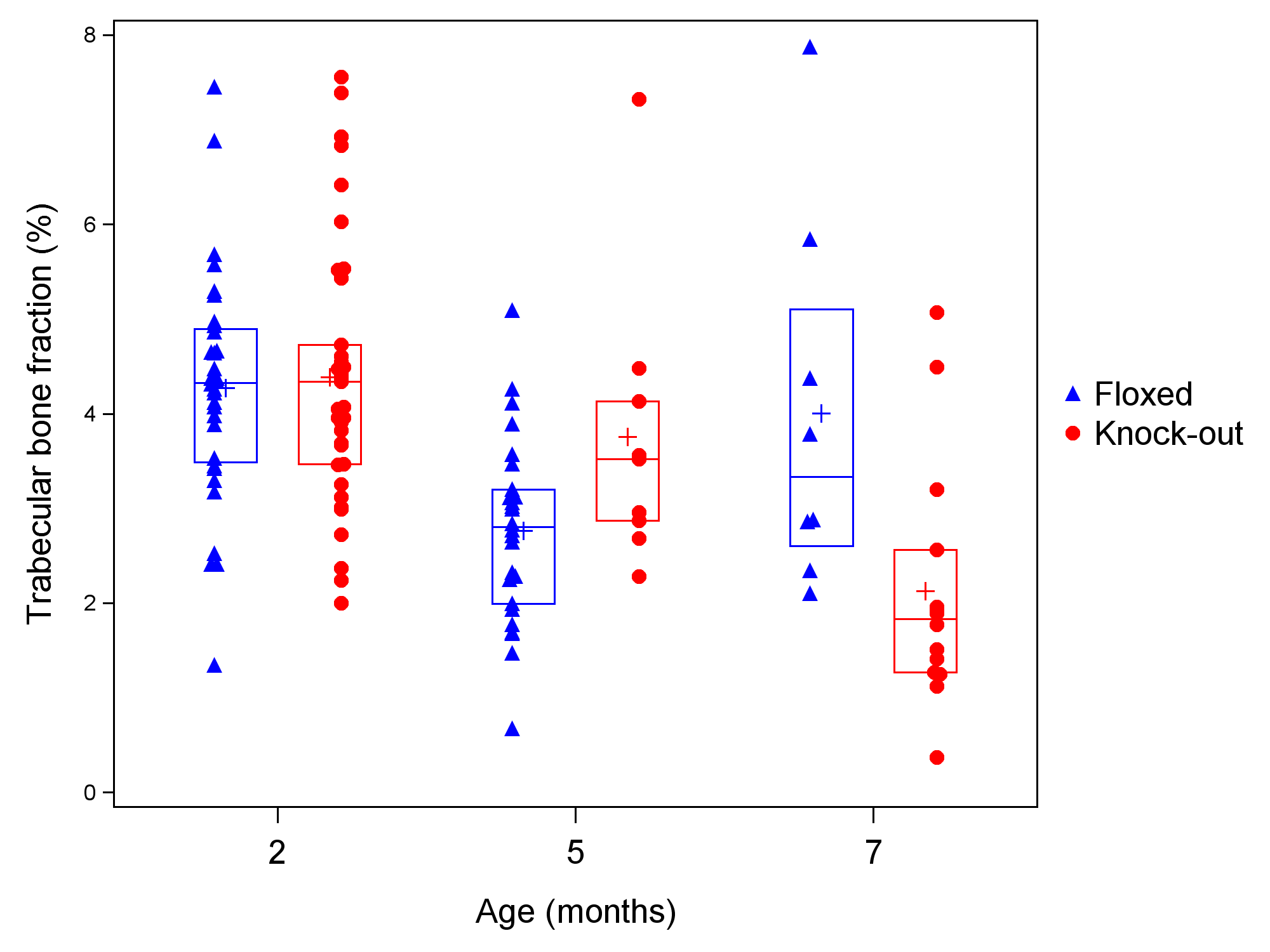

Supplement: S13 Fig — Blue triangles denote Pappa2fl/fl mice and red circles denote Pappa2KO/KO mice. Crosses denote means, while horizontal lines denote the 25th, 50th and 75th percentiles. (TIF) [file pone.0256906.s013.tif]

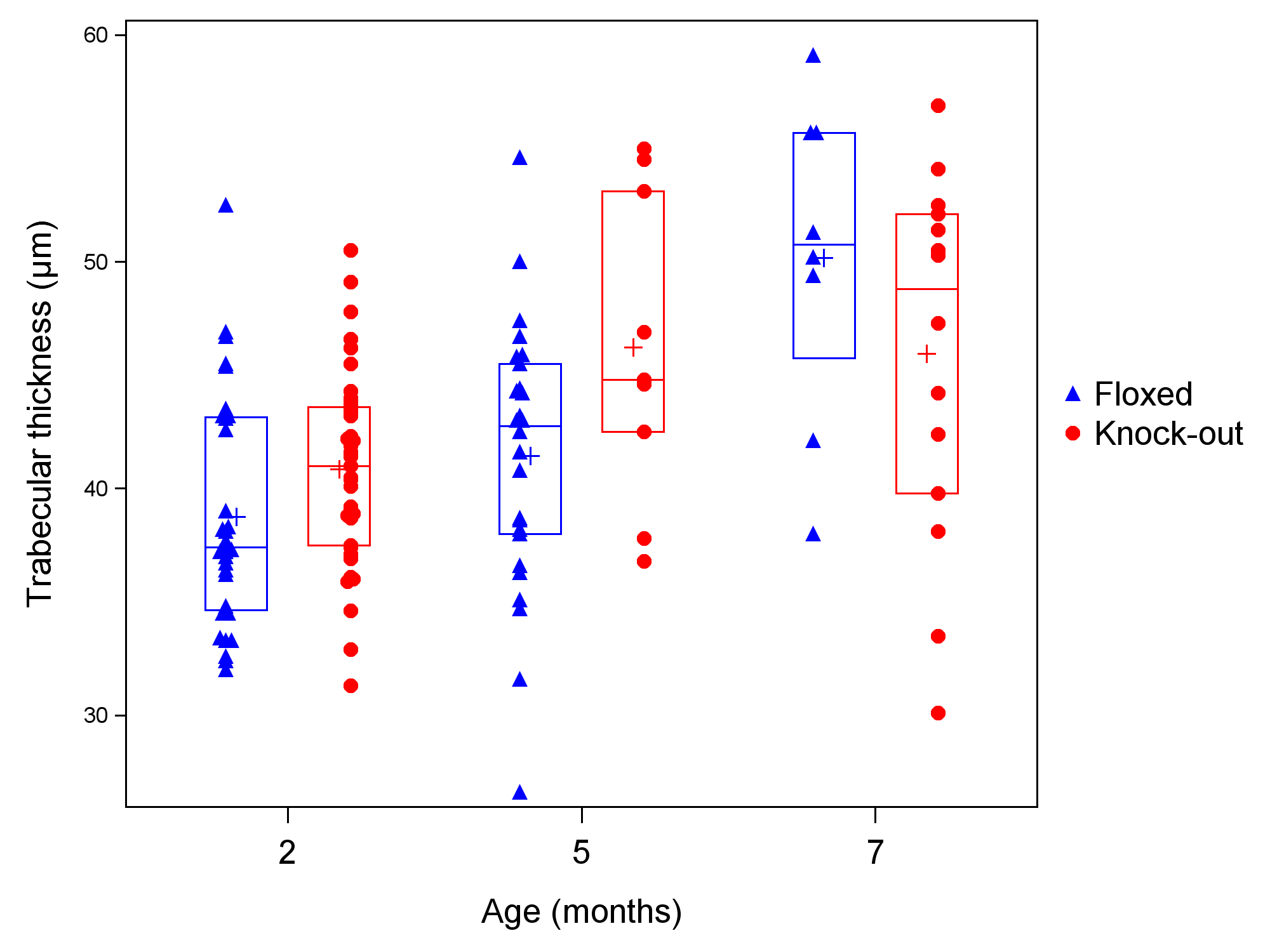

Supplement: S14 Fig — Blue triangles denote Pappa2fl/fl mice and red circles denote Pappa2KO/KO mice. Crosses denote means, while horizontal lines denote the 25th, 50th and 75th percentiles. (TIF) [file pone.0256906.s014.tif]
