# Supplementary material for: Recovery of the maternal skeleton after lactation is impaired by advanced maternal age but not by reduced IGF availability in the mouse
Source: PLoS One. 2021 Sep 1;16(9):e0256906. doi: 10.1371/journal.pone.0256906 (PMC8409645; doi:10.1371/journal.pone.0256906)
Supplement: Attachment — Submitted filename: Response to Reviewers.docx [file pone.0256906.s016.docx]

Dear Dr. Mohan,

Thank you very much for the encouraging response to our manuscript (PONE-D-21-22335), and to the reviewers for their constructive comments. Below, we have outlined our responses to each of the comments, with the reviewer’s comments in italics.

Sincerely,

Julian

**Journal Requirements**

*1. When submitting your revision, we need you to address these additional requirements.*

*Please ensure that your manuscript meets PLOS ONE's style requirements, including those for file naming. The PLOS ONE style templates can be found at*

*https://journals.plos.org/plosone/s/file?id=wjVg/PLOSOne_formatting_sample_main_body.pdf and*

*https://journals.plos.org/plosone/s/file?id=ba62/PLOSOne_formatting_sample_title_authors_affiliations.pdf*

We have revised the title page and file names to be consistent with PLOS ONE style.

*2. Please include further information regarding your in vivo study, per our guidelines (http://journals.plos.org/plosone/s/submission-guidelines#loc-animal-research). Specifically, please provide details regarding:*

*- Source of animals and Description of animal care – Food, water, housing*

*- Animal health monitoring, including frequency and criteria and any efforts made to reduce suffering and distress, such as administering analgesics*

*- whether humane endpoints were in place during the study and how they were applied*

*- the method of euthanasia for the mice*

*- any mortality that occurred outside of planned euthanasia or humane endpoints*

*In addition, as part of your revision, please complete and submit a copy of the Full ARRIVE 2.0 Guidelines checklist, a document that aims to improve experimental reporting and reproducibility of animal studies for purposes of post-publication data analysis and reproducibility: https://arriveguidelines.org/sites/arrive/files/Author%20Checklist%20-%20Full.pdf (PDF). Please include your completed checklist as a Supporting Information file. Note that if your paper is accepted for publication, this checklist will be published as part of your article.*

This information has been added to the manuscript, and we have completed the ARRIVE checklist, revising the manuscript as necessary.

*3. Please note that in order to use the direct billing option the corresponding author must be affiliated with the chosen institute. Please either amend your manuscript to change the affiliation or corresponding author, or email us at plosone@plos.org with a request to remove this option.*

My primary affiliation (SFU) is the same as my chosen institute for direct billing.

**Reviewer #1**

*A very interesting study that shows the role of lactation and after lactation (postweaning) on bone traits during aging. I have two questions about the rationale of the main results:*

*1. If I can understand, authors did not find impairments in cortical bone recovery of Pappa2 deletion mice at 2, 5 and 7 months old (genotype effect). But, I would like to know if authors found genotype*age interaction and single effects of Pappa2 deletion on bone traits at 2, 5 and 7 months in mice collected 3 weeks after weaning compared to the respective Pappa2 floxed control mice and AMC mice. Table 4 did not describe this important question.*

We did not originally include the genotype*age interaction in the model, as the question of whether the effects of genotype per se varied with age was not the focus of our study. However, we have repeated the analyses including this interaction, added the results to Table 4, and described the interaction where significant. The addition of this interaction term did not alter any of the results previously presented.

*2. The effects of breeding and recovery on bone traits were not associated with IGF1 impairments. However, could IGF1 impairment in Pappa2 ko mice be associated with changes in bone traits such as trabecular thickness and cortical area fraction?*

We believe that this is the case and have revised the relevant sentence in the manuscript to read (line 254, in the version with tracked changes):

“The increase in cortical area fraction is consistent with previous work [42], and is likely the result of reduced IGF-I availability, given that it was also observed with a knock-in Pappa2 allele coding for protein that lacked proteolytic activity [52].”

**Reviewer #2**

*Overall, the study is informative and has interesting implications to late pregnancies and breastfeeding in humans.*

*However, there are several concerns that need to be addressed:*

*1. The study lacks mechanistical outcome.*

While our study provides clear evidence of an effect of age on the recovery of the maternal skeleton after lactation, we agree that it does not provide the underlying mechanisms. However, our novel results will motivate further study of the mechanisms; the mechanisms underlying post-lactation recovery are an active area of research, and to some extent remain unknown. Furthermore, our findings that deletion of *Pappa2* does not affect recovery does provide mechanistic insight: IGF-I availability is not crucial for recovery.

*2. Justification of the skeletal site chosen for analysis; Loss of trabecular bone during pregnancy and lactation is often seen in the vertebral body. The trabecular bone at the femur distal metaphysis reduces dramatically between 2-4 months even in virgin mice.*

We have revised the text to provide our rationale (Line 110):

“In mice, the recovery of the vertebrae is complete by 4 weeks post-weaning, whereas the recovery of the femur is incomplete after 3-4 weeks [47,48]. We therefore focused on recovery of the femur 3 weeks after weaning as this skeletal site and time point would allow assessment of variation in recovery.”

We note that a number of other studies in this area examine the femur or tibia without studying the vertebrae (e.g., refs 47, 51, cited in manuscript).

*3. Were calcium levels measured in serum/urine during pregnancy/lactation?*

We did not measure serum or urine calcium levels, consistent with similar studies (e.g., refs 47, 48, 51 cited in manuscript). Studies that do measure calcium levels are generally focused on mechanisms expected to affect calcium metabolism, whereas we are not aware of mechanisms that would link IGF to whole-animal calcium balance. For example, IGF deletion did not affect the response to dietary calcium deprivation (ref 56, cited in manuscript).

*4. What is the predicted mechanism by which cortical bone area reduces after pregnancy/lactation? Endocortical resorption? Any evidence?*

*5. What is the predicted mechanism by which cortical bone area recovers? Periosteal/endosteal apposition?*

We have found little data in the literature to address these issues, but have summarized what is known in the revised manuscript (Line 180):

“The reduction in cortical bone during lactation is due to both reduced periosteal bone formation [52] and increased endocortical bone resorption [53] with the latter being reversed after weaning [54].”

*6. Was the food intake and metabolic rate of the PAPPA2KO dams similar to that of controls?*

Mice were never housed individually, precluding the collection of individual food consumption or metabolic rate. When not breeding, knock-out females were often housed with controls, precluding measurement of average food consumption per genotype. Food intake and metabolic rate are generally not reported in studies of the recovery of the maternal skeleton after lactation (e.g., refs 48 and 51, cited in manuscript), and it is not clear how such data would provide insight into the mechanisms of recovery or the effects of age or genotype.

*While revising your submission, please upload your figure files to the Preflight Analysis and Conversion Engine (PACE) digital diagnostic tool, https://pacev2.apexcovantage.com/. PACE helps ensure that figures meet PLOS requirements.*

Done.
